# Temporal Patterns of Glucagon and Its Relationships with Glucose and Insulin following Ingestion of Different Classes of Macronutrients

**DOI:** 10.3390/nu14020376

**Published:** 2022-01-16

**Authors:** Christian Göbl, Micaela Morettini, Benedetta Salvatori, Wathik Alsalim, Hana Kahleova, Bo Ahrén, Andrea Tura

**Affiliations:** 1 Department of Obstetrics and Gynaecology, Medical University of Vienna, 1090 Vienna, Austria; christian.goebl@meduniwien.ac.at; 2 Department of Information Engineering, Università Politecnica delle Marche, 60131 Ancona, Italy; m.morettini@staff.univpm.it; 3 CNR Institute of Neuroscience, 35127 Padova, Italy; benedetta.salvatori@in.cnr.it; 4 Department of Clinical Sciences, Faculty of Medicine, Lund University, 22184 Lund, Sweden; wathik.alsalim@med.lu.se (W.A.); bo.ahren@med.lu.se (B.A.); 5 Physicians Committee for Responsible Medicine, Washington, DC 20016, USA; HKahleova@pcrm.org

**Keywords:** glucagon determinants, glucagon shape, nutrients, individual associations, glucose, proteins, lipids, mixed meal, glucose homeostasis, mathematical modeling

## Abstract

Background: glucagon secretion and inhibition should be mainly determined by glucose and insulin levels, but the relative relevance of each factor is not clarified, especially following ingestion of different macronutrients. We aimed to investigate the associations between plasma glucagon, glucose, and insulin after ingestion of single macronutrients or mixed-meal. Methods: thirty-six participants underwent four metabolic tests, based on administration of glucose, protein, fat, or mixed-meal. Glucagon, glucose, insulin, and C-peptide were measured at fasting and for 300 min following food ingestion. We analyzed relationships between time samples of glucagon, glucose, and insulin in each individual, as well as between suprabasal area-under-the-curve of the same variables (ΔAUC_GLUCA_, ΔAUC_GLU_, ΔAUC_INS_) over the whole participants’ cohort. Results: in individuals, time samples of glucagon and glucose were related in only 26 cases (18 direct, 8 inverse relationships), whereas relationship with insulin was more frequent (60 and 5, *p* < 0.0001). The frequency of significant relationships was different among tests, especially for direct relationships (*p* ≤ 0.006). In the whole cohort, ΔAUC_GLUCA_ was weakly related to ΔAUC_GLU_ (*p* ≤ 0.02), but not to ΔAUC_INS_, though basal insulin secretion emerged as possible covariate. Conclusions: glucose and insulin are not general and exclusive determinants of glucagon secretion/inhibition after mixed-meal or macronutrients ingestion.

## 1. Introduction

Glucagon is a hormone secreted by the pancreatic alpha cells, and it is a key factor in glucose regulation [1]. In fact, already some decades ago it was shown that glucagon levels increase at low glycemic levels with consequent stimulation of glycogenolysis and gluconeogenesis by the liver, indicating that such glucagon-based counter-regulation mechanisms are crucial for preventing hypoglycemia [2,3,4]. In more recent years, the interest in studying glucagon considerably increased due to the discovery of several actions of metabolic relevance, especially for the maintenance of the glucose homeostasis [1]. As an example, it was suggested that glucagon is involved in the paracrine regulation of the neighboring beta cells: since alpha cells are located in close proximity to the beta cells, it is possible that there is a functional crosstalk between these two islet cell types [1]. In this respect, one study demonstrated that insulin secretion from isolated beta cells was lower than that from beta cells in the presence of alpha cells [5], though other factors produced by the alpha cells may have played a role in the beta-cell function. Other specific studies investigating the role of glucagon in beta-cell function examined isolated islets from mice with genetic deletion of glucagon receptors, showing that glucose-stimulated insulin secretion was impaired [6]. In addition, insulin secretion was overstimulated in mice with beta-cell overexpression of the glucagon receptor [7]. Glucagon was also proved to have hypolipidemic effects, causing decrease in triglyceride and cholesterol, and increase in free fatty acid oxidation [8,9,10,11].

What factors affect glucagon secretion, and its inhibition following food ingestion? It was suggested that glucagon secretion and inhibition are regulated by both intrinsic and paracrine mechanisms, but their relative significance and operating conditions are still partly debated. Furthermore, the intrinsic and paracrine regulation mechanisms are not mutually exclusive: they operate in parallel to control glucagon secretion [12]. At low glycemia, the secretion of glucagon is stimulated by intrinsic mechanisms triggered by glucose, which involve different Na^+^ and Ca^2+^ channels [13,14,15]. When circulating glucose level rises, glucagon secretion is suppressed in healthy individuals, whereas in type 2 diabetes the suppression of glucagon may be impaired [16]. Glucagon suppression is again due to several intrinsic mechanisms, such as alpha-cell membrane depolarization, which determines Na^+^ and Ca^2+^ channels closing [17]. Insulin was the first paracrine factor from the beta cells to show evidence for inhibitory action on glucagon secretion [18]. Some studies also demonstrated that GABA (released as well from the beta cells) can inhibit glucagon secretion by activation of the GABA(A) receptor [19]. Somatostatin, secreted by the pancreatic delta cells, was also proposed as potent inhibitor not only for insulin, but also for glucagon secretion, under hyperglycemic conditions. Indeed, glucagon secretion increases in alpha cells with knockout of the somatostatin receptor 2, this highlighting somatostatin as a mediator for the glucose-based inhibition of glucagon secretion [20,21]. Other studies suggested that glucose can inhibit glucagon secretion by somatostatin-independent pathways [22,23], and it was also reported that glucagon inhibition by insulin may involve stimulation of somatostatin secretion [24]. Furthermore, autonomic nerves, both sympathetic and parasympathetic, may stimulate glucagon secretion [25]. Adrenaline may also stimulate glucagon [26]. As regards incretin hormones, they may have opposite effects: the glucagon-like peptide (GLP-1) inhibits glucagon secretion [27], whereas the glucose-dependent insulinotropic polypeptide (GIP) stimulates it [28], although these effects are dependent on glucose levels.

This large body of studies therefore shows that several factors affect glucagon secretion or suppression. However, despite some still controversial aspects, it can be summarized that the two main factors involved in glucagon regulation (operating through various distinct physiological mechanisms) appear glucose and insulin. In spite of this, the relative contribution of each of the two factors is not fully understood. Especially, it was not elucidated if the contribution of glucose and insulin to glucagon secretion or suppression following food ingestion may depend on the class of the ingested macronutrients. In fact, in some of our previous human studies [29,30], we found differences in the glucagon profiles following separate ingestion of glucose, protein, fat, and a mixture of them. For example, the glucagon release in response to glucose or fat ingestion was reduced compared to the fasting condition, whereas it was increased in response to protein or mixed meal ingestion [30]. However, the degree of association of these changes with glucose and insulin levels was not investigated. In this study, we therefore revisited the data of study [30] to analyze the possible associations of glucagon with glucose and insulin following the ingestion of the indicated macronutrients, both over the entire participants’ cohort and at individual participant’s level. In addition, since we hypothesized that the shape of the hormones temporal curves may influence the investigated associations, we also analyzed the glucagon curves shape following the different macronutrients ingestion.

## 2. Materials and Methods

### 2.1. Participants

In this study, we analyzed 18 participants with normal glucose tolerance (NGT) and 18 patients with type 2 diabetes. The main characteristics of the study participants were described in detail before [30]. Briefly, healthy participants were 11 males and 7 females, had age of 62 ± 5 (mean ± standard deviation) years, and BMI of 25 ± 2 kg/m^2^. Patients with type 2 diabetes were 13 males and 5 females, had age of 63 ± 5 years, and BMI of 27 ± 4 kg/m^2^. They were not under treatment with any pharmacological glucose-lowering therapy. Diabetes duration was 3 ± 2 years. The study was approved by the Ethics Committee in Lund, Sweden (approval number: 2011/361, 23 August 2011). All participants gave written informed consent before the study, which was registered at clinicaltrials.gov (NCT01366794). The study was conducted using good clinical practice and in accordance with the Declaration of Helsinki.

### 2.2. Study Design, Procedures and Measures

This study had randomized crossover design. The 36 participants were studied on 4 occasions, separated by 4–8 weeks, thus for a total of 144 metabolic tests. At each study session, after an overnight fast two baseline blood samples were taken. Then, participants ingested in randomized order one of the following macronutrients: glucose (330 kcal = 83 g; Skåne University Hospital Pharmacy, Lund, Sweden); protein mixture (110 kcal = 30 g; ISO WHEY protein, consisting of milk and egg proteins; Dahlblads Nutrition AB, Stenkullen, Sweden); fat emulsion (110 kcal = 24 mL; Calogen^®^ Neutral, consisting of 50% long-chain triglycerides and 50% water; Nutricia AB, Stockholm, Sweden); the same macronutrients given together as a liquid mixed meal test, with 550 kcal (glucose 330 kcal, protein 110 kcal and fat 110 kcal). The proportions of the macronutrients were selected to represent a common meal with 60% carbohydrate, 20% protein, and 20% fat. Water was ingested simultaneously with each load to standardize the ingested volume to 400 mL. All meals were consumed within 5 min. Blood samples were taken during a 300 min period after each metabolic test. Specifically, glucagon was measured at fasting and at 10, 30, 45, 60, 90, 120, 180, and 300 min following the food ingestion. Glucose, insulin, and C-peptide were measured at five additional time points (5, 20, 75, 150, 240 min).

Glucagon was measured using a recently developed sandwich ELISA, based on monoclonal antibodies against both the C- and N-terminal regions of glucagon, which was shown to have higher specificity and reliability than that of previously used methods [30]. The assay (Mercodia, Uppsala, Sweden; No. 10-1271-01) was therefore specific for pancreatic glucagon and showed 4.4% and 0.8% cross-reactivity with oxyntomodulin and glicentin, respectively, but with no other peptide. Detection limit was 1 pmol/L, whereas intra-assay and interassay coefficient of variation (CV) were 3.3–5.1% and 7.3–9.4%, respectively, ranging from low to high concentrations. Glucose was measured using the glucose oxidase method. Insulin was analyzed using ELISA (Mercodia; No. 10-1113). The sensitivity of the assay was 0.75 pmol/L, with intra-assay and interassay CV of 2.8–4.0% and 2.6–3.6%, respectively. C-peptide was analyzed using double antibody RIA (Merck Millipore, Billerica, MA, USA; No. HCP-20K) based on a specific monoclonal C-peptide antibody. The sensitivity of the assay was 0.02 nmol/L, with intra-assay and interasssay CV of 2.4–9.3% and 6.4%. Further details were reported in our previous study [30].

### 2.3. Calculations

The 0–300 min areas under the curve (AUC) of the variables of interest were calculated using the trapezoid rule (i.e., approximating the region under the graph of the curve by trapezoids, and calculating the sum of their area). The suprabasal component of AUC (ΔAUC) was computed as the AUC subtracted by the fasting value of the variable for the analyzed time interval.

As reported in the original study [30], we exploited appropriate mathematical modeling approach to analyze the metabolic tests. Each test was in fact analyzed by a model yielding the assessment of insulin secretion rate and of different aspects of beta-cell function [31]. In addition, pairs of metabolic tests can be analyzed simultaneously by another model, representing an evolution of the previous beta-cell function model, designed to assess the effect of incretins on insulin secretion (analysis of an oral glucose tolerance test and corresponding isoglycemic intravenous glucose infusion test, taken as reference) [32]. In our previous investigation [30], the glucose test was taken as the reference test, whereas each of the other three metabolic tests (protein, fat, and mixed meal) was the second test in each test pair, analyzed by the model [32]. This approach allowed the assessment of the specific component of insulin secretion potentiation due to fat, protein, and mixed meal, compared to that of glucose alone. In the present investigation, however, for simplicity we calculated the overall potentiation factor in each of the four metabolic tests, including the potentiation induced by glucose, and the potentiation (or depotentiation) due to the different macronutrients.

Specifically, for each macronutrient test, the main model-based parameters analyzed in the present investigation were the beta-cell glucose sensitivity (average slope of the dose-response function, describing the static relationship between insulin secretion and glucose concentration), and the insulin secretion potentiation factor ratio (ratio between the potentiation value at test end and at test beginning). In addition, for a more complete picture of the beta-cell function, in this analysis we also included further parameters not reported previously, i.e., the beta-cell rate sensitivity (marker of early insulin secretion), and the insulin secretion at prescribed glucose reference values (5, 6, 7 mmol/L) [31]. From the model-derived insulin secretion rate, we calculated the total insulin secretion. Insulin clearance was calculated, at each time sample of the metabolic tests, as the ratio between insulin secretion rate and plasma insulin concentration, as recently suggested [33]. The assessment of the main parameters of glucose homeostasis was completed with the calculation of the insulin sensitivity during the metabolic tests, through the ISIcomp index [34].

In this study, we also analyzed the glucagon shape. We classified each glucagon curve according to the number of fluctuations during the test. Precisely, one fluctuation was defined as a change in the trend of the glucagon curve, i.e., a change from an increasing pattern to a decreasing pattern, or the opposite situation. 

### 2.4. Statistical Analyses

AUC, ΔAUC, and basal (fasting) values of the variables of interest, as well as the model-based parameters, were compared among the different metabolic tests by Analysis of Variance (ANOVA) and pairwise comparison, with Benjamini & Hochberg correction for multiple comparisons.

Linear regression analysis was used to assess the possible individual association between glucagon and glucose, and between glucagon and insulin, in each metabolic test for the single subject. Furthermore, in the whole participants’ cohort, we analyzed the association of the suprabasal glucagon AUC (ΔAUC_GLUCA_) with the corresponding variable for glucose and insulin, i.e., ΔAUC_GLU_ and ΔAUC_INS_ (in addition, we considered total insulin secretion and ΔAUC of C-peptide, i.e., ΔAUC_CP_). This analysis was accomplished by regression performed through linear mixed-effect model approach, whereby the participant’s identification number was included as a random effect. In this analysis, we had to report a modified R^2^ (marginal R^2^). We also performed stepwise regression analysis to determine possible further determinants of ΔAUC_GLUCA_ other than ΔAUC_GLU_ and ΔAUC_INS_, thus yielding an optimal model based on minimization of the Akaike’s information criterion (AIC).

Chi-square test, with Yates’s continuity correction, was used to assess possible differences in categorical data. Precisely, it was used to assess possible differences in the number of significant regressions between glucagon and glucose on one side and glucagon and insulin on the other side, as well as in the number of the indicated significant regressions among the different metabolic tests. Chi-square test was also used in the shape analysis to assess differences in the number of glucagon curve fluctuations.

The statistical distribution of any continuous variable was assessed, and natural logarithmic transformation was performed in the case of skewed distribution before statistical testing. The two-sided significance level was set at 5% (*p* < 0.05). Values are reported as mean ± standard error (SE) unless otherwise specified. Statistical analysis was performed with R (version 3.6.3, The R Foundation for Statistical Computing, Vienna, Austria), and contributed packages.

## 3. Results

### 3.1. Plasma Concentrations of Glucagon, Glucose, Insulin and C-Peptide

Fasting values, AUC and ΔAUC for plasma glucagon, glucose, insulin, and C-peptide in the whole participants’ cohort after the mixed meal and the single macronutrients tests are reported in Table 1.

Fasting values for glucagon, glucose and C-peptide were not different among macronutrients tests, whereas those for insulin showed some differences, suggesting not negligible intrasubject variability in terms of basal insulin secretion and/or insulin clearance. At converse, AUCs were all different (*p* ≤ 0.0004). Similarly, ΔAUCs were typically different among tests (*p* ≤ 0.04), though not all differences reached statistical significance for ΔAUC_GLU_ and ΔAUC_CP_. 

As regards ΔAUC_GLUCA_, in more details, it was positive in 86 out of the 144 total number of tests (32, 9, 34, 11, in mixed meal, glucose, protein, fat tests, respectively), and it was negative in the remaining 58 cases (4, 27, 2, 25, respectively). Thus, ΔAUC_GLUCA_ was mainly positive in meal and protein tests, whereas it was mainly negative in glucose and fat tests. Of note, the proportion of positive vs. negative cases in each metabolic test was found significantly different, according to the chi-square test (*p* ≤ 0.002). On average, ΔAUC_GLUCA_ was therefore negative for glucose and fat tests (lower in the former), and positive for mixed meal and protein tests (higher in the latter; see Table 1). As expected, based on the illustrated findings, ΔAUC_GLUCA_ was markedly different between each pair of tests (*p* ≤ 0.0003), except for glucose and fat tests comparison, where the difference was slight (*p* = 0.04). 

### 3.2. Insulin Secretion/Beta-Cell Function, Insulin Sensitivity, Insulin Clearance

Insulin secretion, beta-cell function, insulin sensitivity and insulin clearance are reported in Table 2. Total insulin secretion was different among the four metabolic tests (*p* ≤ 0.01). Precisely, it was higher in the mixed meal test, then progressively lower in glucose, protein, and fat tests. Fasting insulin secretion essentially reflected the differences observed in plasma insulin.

Beta-cell function parameters showed somehow heterogeneous behavior, as glucose sensitivity was typically different among tests (*p* ≤ 0.02, except for mixed meal vs. glucose comparison), with the same progressive variation from mixed meal test to fat test as observed for insulin secretion. The potentiation factor ratio showed instead less significant differences, and the rate sensitivity was typically similar among tests, with only one comparison showing significant difference (mixed meal vs. fat tests). Insulin secretion at reference glucose values showed again heterogeneous results, as it was different in the majority of comparisons at 7 mmol/L (*p* ≤ 0.004), whereas less differences were shown at 5 and 6 mmol/L.

Insulin sensitivity (ISIcomp) was markedly different in almost all comparisons (*p* < 0.0001), with only mixed meal vs. glucose test not showing significant difference. It was higher in fat, then progressively decreased in protein, glucose, and meal. Insulin clearance, as average value during the 300 min interval of the metabolic tests, was different in all comparisons (*p* ≤ 0.01), with the same sequence of magnitude shown by ISIcomp.

### 3.3. Individual Associations in All Tests of Glucagon vs. Glucose, Insulin, C-Peptide, and Insulin Secretion

In regression analysis in the single participants, glucagon showed significant direct relationship with glucose in 18 cases, out of the 144 total number of cases (Table 3). Direct relationship with insulin was observed in a much higher number of cases (60 cases, *p* < 0.0001). Inverse relationship between glucagon and glucose was observed in only 8 cases, and similarly for glucagon and insulin (5 cases). R^2^ values (mean ± SE and range) are reported in Table 3.

Since C-peptide is a marker of insulin secretion that may be more accurate than insulin itself (it is secreted equimolarly, and does not undergo hepatic extraction), we also considered the relationships between glucagon and C-peptide. However, there was no relevant improvement in terms of number of cases showing significant relationship (Table 3). Similar considerations hold for the model-derived insulin secretion. Therefore, in the following analyses, we further analyzed the possible relationship of glucagon with glucose and insulin only.

### 3.4. Individual Associations of Glucagon vs. Glucose and Insulin for the Different Macronutrients

**Figure 1:**
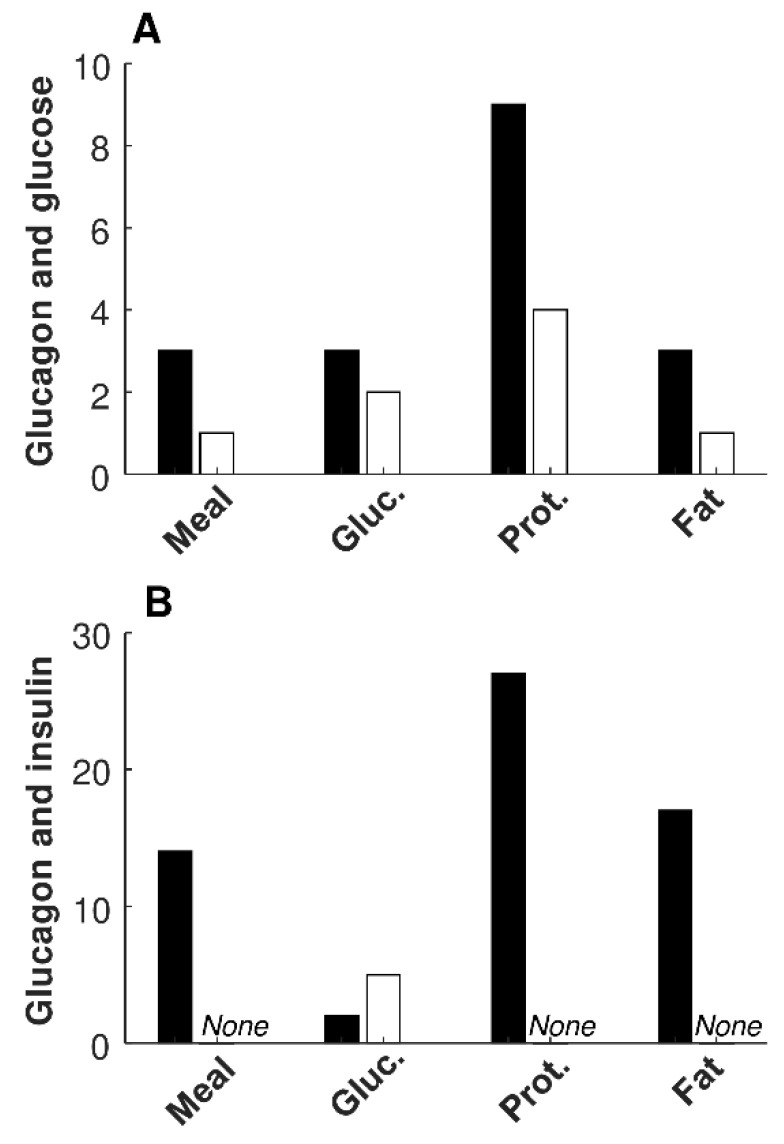
Number of cases showing significant direct regression (left bar, black) and inverse regression (right bar, white) between glucagon and glucose (**A**), and between glucagon and insulin (**B**), for different metabolic tests in individual regression analysis.

Information on significant relationships between glucagon and glucose, and between glucagon and insulin, in each of the four metabolic tests, are reported in Table 4. The number of significant direct regressions of glucagon with glucose was lower than that with insulin for mixed meal, protein, and fat tests (*p* ≤ 0.006); thus, it was not different only for the glucose test. No differences were observed in the number of significant inverse regressions in any of the tests, due to the low number of such regressions. Figure 1 reports a bar graph summarizing the number of significant regressions between glucagon and glucose (Figure 1 A), as well as between glucagon and insulin (Figure 1 B), for the different metabolic tests. The few significant inverse regressions between glucagon and insulin were all observed in the glucose test (Figure 1 B; Table 4). 

**Figure 2:**
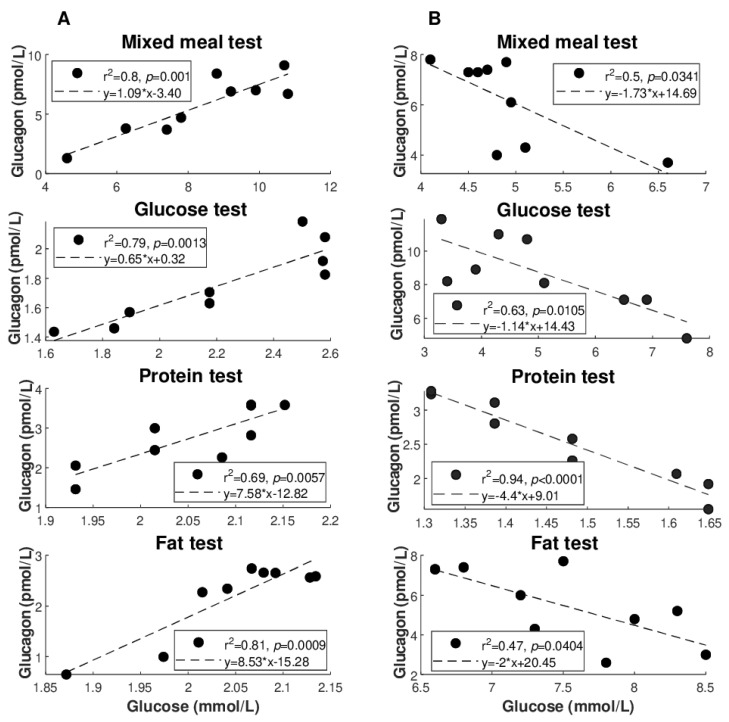
Best regression (highest R^2^ value) between glucagon and glucose for each of metabolic tests in individual regression analysis ((**A**) direct regressions; (**B**) inverse regressions). Values are log-transformed in following cases: glucose, protein, and fat tests in (**A**); protein test in (**B**).

**Figure 3:**
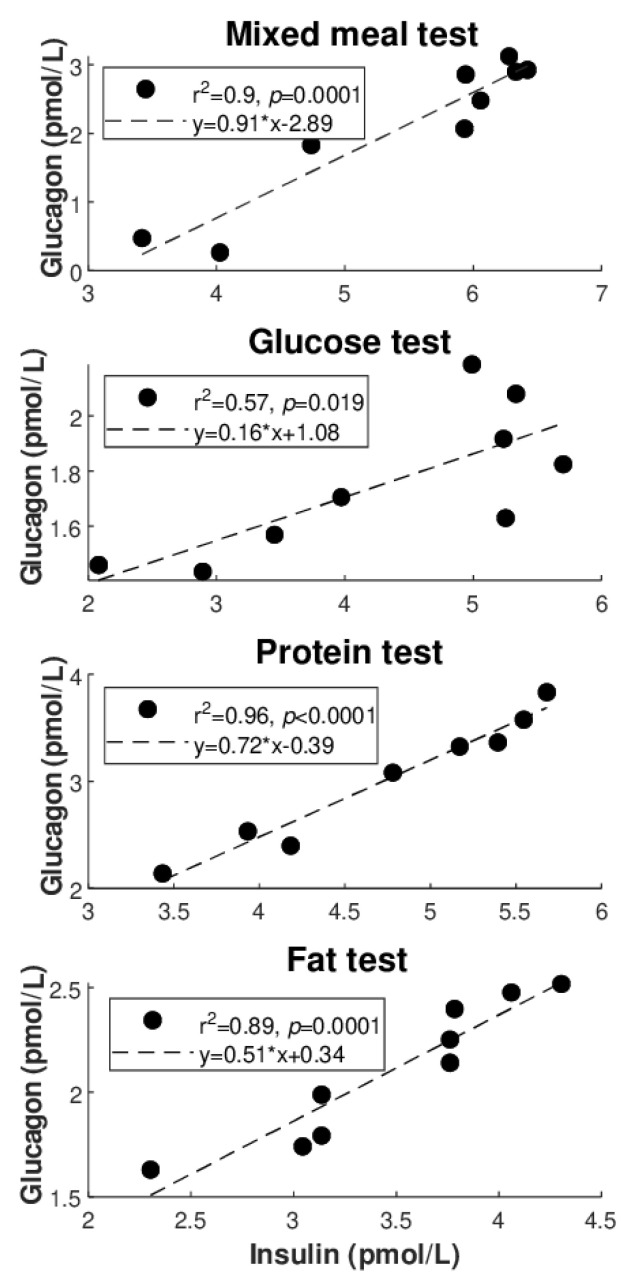
Best regression (highest R^2^ value) between glucagon and insulin for each metabolic test in individual regression analysis (direct regressions). All values are log-transformed.

Figure 2 illustrates the best regression (highest R^2^ value: see also Table 4) between glucagon and glucose for each of the metabolic tests (Figure 2A: direct regressions; Figure 2B: inverse regressions). Similarly, Figure 3 illustrates the best regression between glucagon and insulin for each metabolic test, but only limited to the direct regressions. Based on these figures, the degree of individual association between glucagon and insulin, though not frequently, may reach strong values (up to R^2^ = 0.96), whereas somehow less strong values are seen for glucagon and glucose (R^2^ = 0.81 as maximum, at least for direct regressions).

We also tested whether the number of significant regressions of glucagon vs. glucose, and vs. insulin, is different among the four metabolic tests. For the number of significant direct regressions between glucagon and insulin, we found difference in each comparison, i.e., in each pair of metabolic tests compared (*p* ≤ 0.03), except for mixed meal vs. fat test. We did not detect further significant differences in the other statistical tests of this specific analysis, likely due to the generally low number of cases (see again Table 4).

Furthermore, we tested possible differences in the number of significant regressions pooling the participants for the glucose tolerance (NGT (*N* = 18), type 2 diabetes, (*N* = 18)), for the degree of obesity (lean for BMI < 25 kg/m^2^ (*N* = 15), overweight/obese otherwise (*N* = 21)), and for sex (males (*N* = 24), females (*N* = 12)). However, no difference reached statistical significance in this analysis (*p* ≥ 0.07). 

### 3.5. Associations between Glucagon, Glucose, and Insulin in Analyses over the Whole Subjects’ Cohort

In the regression analysis between ΔAUC_GLUCA_ and ΔAUC_GLU_ over the whole participants’ cohort, we found a significant but nonetheless weak inverse relationship, as indicated by the very low marginal R^2^ (R^2^ = 0.04, *p* = 0.019). When considering ΔAUC_GLUCA_ and ΔAUC_INS_, the relationship was not significant. In multivariable regression, with both ΔAUC_GLU_ and ΔAUC_INS_ as independent variables, we confirmed the results of the previous analysis: in fact, ΔAUC_GLU_ remained significant (*p* = 0.021), whereas ΔAUC_INS_ remained not significant, with marginal R^2^ virtually identical. 

Subsequently, we introduced the type of metabolic test as categorical variable (i.e., four levels variable: mixed meal, glucose, protein, fat), but it was not found as significant covariate, and in addition it determined loss of significance for ΔAUC_GLU_. We also tested the effect of the different categorical variables previously mentioned: (*i*) glucose tolerance (levels: NGT, type 2 diabetes); (*ii*) obesity (levels: lean, overweight/obese); (*iii*) sex (levels: males, females). In all three analyses, the categorical variable never emerged as significant covariate, and in the case (*ii*) and (*iii*) it also determined loss of significance for ΔAUC_GLU_, whereas its significance was maintained in (*i*) (*p* = 0.032). 

We also performed stepwise regression analysis, with minimal statistical model including both ΔAUC_GLU_ and ΔAUC_INS_, with the aim of analyzing what possible variables may contribute to explain ΔAUC_GLUCA_ and investigating whether such further variables may cause ΔAUC_INS_ to emerge as significant covariate. In the initial model, in addition to ΔAUC_GLU_ and ΔAUC_INS_, we included basal (fasting) and total insulin secretion, beta-cell glucose and rate sensitivity, potentiation factor ratio, insulin sensitivity (ISIcomp), and average insulin clearance. We also included all the categorical variables previously described, i.e., metabolic test, glucose tolerance, obesity, sex. In the final model, in addition to ΔAUC_GLU_ and ΔAUC_INS_ included by default (minimal model), the stepwise procedure selected basal insulin secretion, metabolic test, and glucose tolerance, with final R^2^ (adjusted) of 0.30 and *p* < 0.0001, and with slight improvement (decrease) of the Akaike’s AIC equal to 4.7%, compared to that of the initial model. However, ΔAUC_INS_ did not gain significance, and ΔAUC_GLU_ lost significance as well, but the role of insulin somehow emerged in terms of insulin secretion at basal (showing inverse relationship with ΔAUC_GLUCA_). In the stepwise regression with only ΔAUC_GLU_ as minimal model, the stepwise procedure included the same variables as before, with similar values for adjusted R^2^, *p*, and AIC. Of note, ΔAUC_GLU_ was again not significant. Consistently, without any minimal model, neither ΔAUC_GLU_ nor ΔAUC_INS_ were selected by the stepwise procedure, which again selected only basal insulin secretion, and metabolic test plus glucose tolerance as categorical covariates (adjusted R^2^ = 0.31, *p* < 0.0001, AIC reduction of 6.3%). If basal insulin secretion is replaced by insulin secretion at reference glucose value (see Table 2), in the case of glucose at 5 or 6 mmol/L (5–6 mmol/L being the typical range of basal glycemia in nondiabetic people), again the stepwise procedure selects such insulin secretion parameter for the optimal model. In this case, only metabolic test is selected as further covariate.

### 3.6. Shape Analysis of the Glucagon Curves

**Figure 4:**
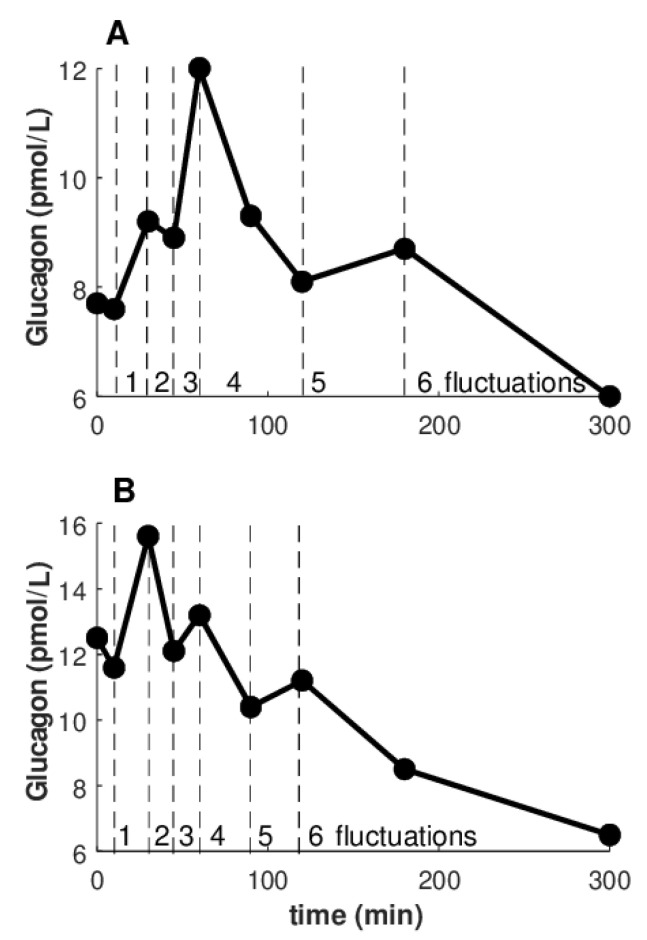
Calculation of fluctuations in glucagon curve: examples of cases with highest number of fluctuations observed (6 fluctuations). **A**: curve with positive ΔAUC_GLUCA_; **B**: curve with negative ΔAUC_GLUCA_.

Figure 4 illustrates how fluctuations were calculated. Precisely, we illustrated two of the few cases with the highest number of fluctuations that were observed (6 fluctuations). However, in the majority of cases, the glucagon curves showed from 1 to 5 fluctuations (Table 5). 

We found some significant differences comparing the number of curves with increasing number of fluctuations. For the glucagon curves characterized by positive ΔAUC_GLUCA_, differences were detected comparing 1 vs. 2 fluctuation curves, as well as 1 vs. 5, 2 vs. 3, 3 vs. 5 (*p* ≤ 0.01). Of note, 3 fluctuations and 1 only fluctuation were the most common curve shapes (26 and 25 curves out of 86 curves, respectively). For the glucagon curves with negative ΔAUC_GLUCA_, differences were between 1 vs. 2, 1 vs. 3, 3 vs. 4, and 3 vs. 5 fluctuations (*p* ≤ 0.03). The most common curve shape was 3 fluctuations (20 of 58 curves). When considering the four metabolic tests separately, significant differences in the number of the fluctuations were rarer (likely partially due to lower statistical power). Curiously, only in the protein test, for the glucagon curves with positive ΔAUC_GLUCA_ several significant differences were observed: 1 fluctuation vs. all other shape types except for 3 fluctuations (*p* < 0.0001), and 3 fluctuations again vs. all other shape types except obviously for 1 fluctuation (*p* ≤ 0.01).

## 4. Discussion

In this study, we analyzed the possible associations of glucagon with glucose and insulin following ingestion of different macronutrients. The main aim was to investigate the relative contribution of glucose and insulin as determinants of glucagon release or inhibition following food ingestion. Precisely, we aimed to evaluate if glucose and insulin relationships with glucagon may depend on the type of macronutrient ingested. To our knowledge, this is the first study analyzing the associations of glucose and insulin with glucagon in relation to all three main classes of macronutrients, in isolation and in combination, i.e., glucose, proteins, lipids, and the mixed meal. 

We found that the combined ingestion of the macronutrients (mixed meal), and the simple protein ingestion, typically determine stimulation of glucagon release compared to the fasting condition, rather than inhibition. In contrast, for glucose and fat ingestion, we observed that glucagon inhibition was more frequent than stimulation, though stimulation was still observed not rarely. When analyzing the relationship of glucagon with glucose and with insulin in each participant, and for each metabolic test, for glucose we found a modest number of significant associations, both direct and inverse (18 and 8 out of the 144 total metabolic tests, respectively). For insulin, more cases were observed, at least in terms of direct relationship (60 direct and 5 inverse). Interestingly, the frequency of direct relationship of glucagon with glucose, and with insulin as well, was typically different among the four metabolic tests, whereas significant differences among tests were not reached for the inverse relationship, likely due to the low number of cases. These findings suggest that, although glucose and insulin are typically considered as the main determinants of glucagon release or inhibition, acting with several distinct mechanisms [1,15,24,27], the relevance of both glucose and insulin role may be somehow less strong than generally believed (especially glucose). Therefore, it cannot be excluded that other factors, independent (at least partially) from glucose and insulin levels, may have a role comparable to that of glucose or insulin in determining stimulation or inhibition of glucagon release. In addition, our findings suggest that the relative contribution of the glucagon determinants following food ingestion may be largely dependent on the class of nutrients ingested, since strongly heterogeneous results were observed for the analyzed four types of meals. Thus, amino acids may be other glucagon determinants, at least after mixed meal or protein ingestion. In addition, further candidate glucagon determinants may be those related to autonomic stimulation, as briefly mentioned previously [25]. 

To corroborate the findings obtained in single individuals, we performed regression analyses over the whole participants’ cohort. These analyses, though not totally in agreement with the individual analyses (i.e., role of insulin here found even weaker than that of glucose), overall confirmed that the contribution of glucose and insulin in determining glucagon levels may be limited, and, especially, possibly dependent on the type of macronutrient ingested. This was in fact suggested by the inclusion of the metabolic test categorical variable in the optimal model for the prediction of the glucagon levels. Of note, in these analyses a role emerged for basal rather than total insulin secretion, despite one may have expected the latter rather than the former. This may imply a more important role for basal insulin secretion as regulator of the alpha cell than the dynamic change in insulin after an oral challenge. However, the present study should be considered as the basis for future studies, aimed to determine, ideally, all factors involved in glucagon release and inhibition, and their relative contribution in relation to the type of food ingested. Indeed, since glucagon has progressively emerged as a crucial contributor of glucose homeostasis, accurate identification of the effects on its release or inhibition of different types of dietary habits (i.e., based on different relative composition of the nutrients) may be relevant for the prevention of type 2 diabetes (or to avoid further metabolic derangement in people already with diabetes). The importance of precision nutrition in metabolic disturbances, including type 2 diabetes, was recently emphasized in the general context of precision diabetes medicine [35,36]. Thus, a deep understanding of the dietary habits regarding the effects on glucagon may be beneficial, possibly being of help for the identification of the most appropriate individualized diet prescriptions, specifically tailored for the single patient.

Limited comparison is possible with previous studies. In our previous study [30], based on the same participants’ cohort of the current investigation, the analysis of glucagon was not among the main aims. Glucagon was analyzed, but mainly in terms of differences among the metabolic tests, with no analysis of its associations with other variables, which instead was the focus of this new study. In an earlier study of our research group [29], with again administration of different macronutrients, some glucagon information was again reported, but similar considerations as for the study [30] hold. In the study by Yabe D. et al., [37], glucagon was investigated in both nondiabetic and type 2 diabetes participants, similarly to our study. For each participant, an oral glucose tolerance test and a mixed meal test was performed. Significant relationships were found between the area under the curve of glucose and that of glucagon and insulin for specific time intervals. This agrees with the findings of our AUC-based analyses in terms of significant relationships between glucagon and glucose, whereas we did not find relationships with insulin, differently to the study [37]. This partial disagreement may be due to several reasons, among which the duration of the metabolic tests (120 min only vs. 300 min in our tests), and the methodology to perform the regression analyses. In fact, in study [37] the emphasis was on the possible effects of glucagon and insulin on glucose, rather than the effects of glucose and insulin on glucagon, which is the focus of our current study. This affects the role assigned to independent and dependent variables in the regression analysis, and results can be influenced accordingly. In study [37], AUC-based analyses were not corroborated by the individual analyses.

In this study, we also analyzed glucagon shape, as this may provide complementary information to the findings from the regression analyses. In fact, the relevance of the shape analysis in metabolism was recently emphasized in several studies, at least for the glucose curve (see for instance the review study [38]). However, shape analyses were found of importance even for the study of the insulin and C-peptide curves [39], and hence we hypothesized that the analysis of glucagon may have provided relevant information. We found that the glucagon curves in our dataset are extremely heterogeneous, showing from few to many fluctuations (up to a number of fluctuations almost equal to the maximum observable, in relation to the number of time samples collected). This further underlies the heterogeneous behavior of the glucagon kinetics following food ingestion. In addition, since the frequency of the different shape categories was not always similar among the different types of metabolic tests, this further corroborated the conclusions previously illustrated in terms of heterogeneity of glucagon behavior, for the different macronutrients. To our knowledge, no further study quantitatively analyzed the glucagon curves shape, and this is another novelty of our study.

In one of our previous studies [40], we observed that during an oral glucose tolerance test the association between glucagon and insulin appears more evident when considering plasma C-peptide rather than plasma insulin. As mentioned previously, this may be due to the reason that plasma C-peptide could be a marker of insulin secretion more accurate than plasma insulin, since C-peptide is secreted equimolarly with insulin, but at difference with insulin it does not undergo hepatic extraction. Since hepatic extraction can vary among subjects and may also show temporal variations in the single individual during a metabolic test, this may act as confounding factor for the role of insulin in the analysis of the relationships with other hormones, such as glucagon. For this reason, in our first set of regression analyses, we also considered C-peptide, as well as model-based insulin secretion, which again is derived by plasma C-peptide rather than plasma insulin. However, in this study we did not appreciate relevant advantages in using C-peptide (or C-peptide-based insulin secretion) in place of insulin. Therefore, the subsequent analyses were performed in fact with insulin. The study [40] proposed a model for the assessment of the sensitivity of glucagon to insulin inhibition. However, the present study revealed that the application of such modeling approach may not be always appropriate, since in many cases the individual relationship between glucagon and insulin appears missing. Thus, in future studies, the model of study [40] may still be applied, but only to those patients/tests where the glucagon-insulin relationship is present.

The sample size of this study was not large, and this should be acknowledged as a limitation. On the other hand, such sample size appears reasonable in relation to the complex experimental protocol, requiring four metabolic tests in separate days for each participant, and including healthy people that are typically more difficult to recruit, especially in elaborate and time-demanding study protocols, as in the present investigation (with each test lasting five hours). In relation to the long duration of each metabolic test, and the large number of time samples per test, we can claim that the analyses in the single individuals are robust. Instead, it is correct to acknowledge that in the analyses over the whole cohort a larger sample size would have been beneficial; in fact, it cannot be excluded that the weak relationships between glucagon, glucose, and insulin, observed in such whole cohort analyses, may improve somehow when studying larger datasets. This advocates for the need of future studies in the field. In relation to the sample size issue, in the whole cohort analyses we did not perform separate investigations for the different groups of participants (i.e., NGT and type 2 diabetes participants, as well as lean and overweight/obese, male, and female participants), to limit the risk for critical loss of statistical power in the analyses. Nonetheless, the role of the indicated participants’ categories was considered by introducing appropriate categorical variables, as previously described.

In conclusion, in metabolic tests based on the administration of different macronutrients, we analyzed the associations between glucagon, glucose and insulin, while also considering in some analyses the possible contribution of insulin sensitivity and secretion, beta-cell function, and insulin clearance. We also assessed the shape of the glucagon curves. We found that the glucagon time patterns are highly heterogeneous, and the relationship with glucose and insulin is typically not strong, except in few individual cases. This suggests that the role of glucose and insulin as determinants of glucagon release or inhibition following food ingestion is likely less strong than often suggested in previous investigations, and in addition it may strongly depend on the class of nutrients ingested. Further studies are needed to identify in detail all main determinants of glucagon release/inhibition and their relative relevance depending on the food composition. This may be important for the appropriate assessment of the effects of different diet regimes on glucagon and may contribute to the identification of the optimal dietary strategy, likely varying among individuals, for delaying or preventing the onset of type 2 diabetes, or for preventing worsening of dysglycemia in people already suffering from diabetes.

## Figures and Tables

**Table 1:** Fasting values, area under the curve (300 min), and related suprabasal component (AUC and ΔAUC, respectively) for plasma glucagon, glucose, insulin, and C-peptide, after ingestion of a mixed meal or single macronutrients in a cohort of 36 subjects (18 subjects with normal glucose tolerance and 18 subjects with type 2 diabetes). Data are mean ± SE.

	Mixed Meal	Glucose	Protein	Fat
Fasting values				
Glucagon (pmol/L)	6.99 ± 0.53	7.94 ± 0.82	7.64 ± 0.62	7.36 ± 0.55
Glucose (mmol/L)	6.23 ± 0.25	6.43 ± 0.26	6.18 ± 0.20	6.20 ± 0.16
Insulin (pmol/L)	45.32 ± 6.22	47.44 ± 5.23	42.06 ± 5.81 ^3^	35.32 ± 5.51 ^4,5^
C-peptide (pmol/L)	0.50 ± 0.04	0.49 ± 0.04	0.50 ± 0.05	0.44 ± 0.04
AUC values				
Glucagon (pmol/L·h)	50.87 ± 3.51	25.34 ± 1.78 ^1^	72.24 ± 4.39 ^2,3^	32.78 ± 2.22 ^4,5,6^
Glucose (mmol/L·h)	36.40 ± 1.78	38.67 ± 1.74 ^1^	30.65 ± 0.91 ^2,3^	31.90 ± 0.81 ^4,5,6^
Insulin (pmol/L·h)	1362.9 ± 195.7	839.7 ± 109.9 ^1^	340.2 ± 48.1 ^2,3^	142.8 ± 23.8 ^4,5,6^
C-peptide (pmol/L·h)	6931 ± 508	6244 ± 492 ^1^	3127 ± 265 ^2,3^	2110 ± 186 ^4,5,6^
ΔAUC values				
Glucagon (pmol/L·h)	17.58 ± 2.66	−11.02 ± 2.77 ^1^	35.54 ± 3.49 ^2,3^	−1.39 ± 1.38 ^4,5,6^
Glucose (mmol/L·h)	5.26 ± 0.97	6.52 ± 1.08	−0.27 ± 0.52 ^2,3^	0.89 ± 0.51 ^4,5^
Insulin (pmol/L·h)	1136.3 ± 169.2	602.5 ± 90.8 ^1^	129.9 ± 31.6 ^2,3^	−33.8 ± 8.9 ^4,5,6^
C-peptide (pmol/L·h)	2118.3 ± 420.7	3819.4 ± 365.8 ^1^	553.1 ± 143.4 ^2,3^	−81.3 ± 65.0 ^4,5^

^1^ *p* < 0.05 between Glucose and Mixed Meal; ^2^ between Protein and Mixed Meal; ^3^ between Protein and Glucose; ^4^ between Fat and Mixed Meal; ^5^ between Fat and Glucose; ^6^ between Fat and Protein.

**Table 2:** Main metabolic parameters after ingestion of a mixed meal or single macronutrients in a cohort of 36 subjects (18 subjects with normal glucose tolerance and 18 subjects with type 2 diabetes). Data are mean ± SE.

	Mixed Meal	Glucose	Protein	Fat
Insulin secretion				
Fasting insulin secretion (pmol·min·m^−2^)	61.31 ± 5.09	60.84 ± 5.32	59.30 ± 5.68	54.22 ± 4.90 ^4,5^
Total insulin secretion (nmol·m^−2^)	53.24 ± 4.15	47.29 ± 3.91 ^1^	23.07 ± 2.03 ^2,3^	15.60 ± 1.42 ^4,5,6^
Beta-cell function				
Glucose sensitivity (pmol min^−1^·m^−2^·mM^−1^)	54.93 ± 7.69	46.17 ± 9.64	35.40 ± 6.17 ^2,3^	7.81 ± 2.94 ^4,5,6^
Rate sensitivity (pmol·m^−2^·mM^−1^)	535.7 ± 176.9	199.7 ± 44.0	512.1 ± 190.8	181.6 ± 68.5 ^4^
Potentiation factor ratio (unitless)	1.46 ± 0.15	1.28 ± 0.15	1.15 ± 0.39 ^2,3^	0.76 ± 0.05 ^4,5^
Insulin secretion at reference glucose				
At. 5 mmol/L (pmol·min·m^−2^)	79.96 ± 10.10	63.56 ± 6.62	40.49 ± 4.58 ^3^	43.78 ± 5.28 ^5^
At. 6 mmol/L (pmol·min·m^−2^)	128.91 ± 13.94	107.00 ± 11.66	60.43 ± 6.81 ^2,3^	50.48 ± 4.91 ^4,5^
At. 7 mmol/L (pmol·min·m^−2^)	181.25 ± 20.06	152.88 ± 20.51	91.39 ± 9.85 ^2,3^	58.37 ± 5.95 ^4,5,6^
Other metabolic parameters				
Insulin sensitivity, ISIcomp (unitless)	7.74 ± 1.00	8.68 ± 1.20	17.39 ± 2.36 ^2,3^	31.22 ± 4.21 ^4,5,6^
Insulin clearance (average) (L·min^−1^·m^−2^)	1.32 ± 0.30	1.37 ± 0.17 ^1^	1.81 ± 0.20 ^2,3^	3.14 ± 0.45 ^4,5,6^

^1^ *p* < 0.05 between Glucose and Mixed Meal; ^2^ between Protein and Mixed Meal; ^3^ between Protein and Glucose; ^4^ between Fat and Mixed Meal; ^5^ between Fat and Glucose; ^6^ between Fat and Protein.

**Table 3:** Number of significant relationships (direct and inverse) between glucagon and glucose, glucagon, and insulin, as well as C-peptide and insulin secretion. R^2^ values are also reported.

	Direct Relationship	Inverse Relationship
Glucagon vs. glucose		
*N* (%, n. on total)	12.5 (18/144)	5.6 (8/144)
R^2^ (Mean ± SE)	0.62 ± 0.02	0.62 ± 0.03
R^2^ (Min–Max)	0.45–0.81	0.47–0.94
Glucagon vs. insulin		
*N* (%, n. on total)	41.7 (60/144)	3.5 (5/144)
R^2^ (Mean ± SE)	0.71 ± 0.02	0.60 ± 0.02
R^2^ (Min–Max)	0.47–0.96	0.50–0.75
Glucagon vs. C-peptide		
*N* (%, n. on total)	34.0 (49/144)	6.3 (9/144)
R^2^ (Mean ± SE)	0.68 ± 0.03	0.63 ± 0.03
R^2^ (Min–Max)	0.44–0.96	0.46–0.88
Glucagon vs. ins. secr.		
*N* (%, n. on total)	28.5 (41/144)	2.8 (4/144)
R^2^ (Mean ± SE)	0.63 ± 0.02	0.57 ± 0.01
R^2^ (Min–Max)	0.45–0.89	0.51–0.67

**Table 4:** Number of significant relationships (direct and inverse) between glucagon and glucose, and glucagon and insulin, in different metabolic tests. R^2^ values are also reported.

	Mixed Meal		Glucose		Protein		Fat	
	Direct Relation.	Inverse Relation.	Direct. Relation.	Inverse Relation.	Direct Relation.	Inverse Relation.	Direct Relation.	Inverse Relation.
Glucagon vs. glucose								
*N* (%, number on total) 8.3 (3/36)		2.8 (1/36)	8.3 (3/36)	5.6 (2/36)	25.0 (9/36)	10.2 (4/36)	8.3 (3/36)	2.8 (1/36)
R^2^ (Mean ± SE) 0.69 ± 0.03		-	0.70 ± 0.02	-	0.58 ± 0.02	0.72 ± 0.03	0.60 ± 0.03	-
R^2^ (Min–Max) 0.53–0.80		-	0.59–0.79	0.49–0.63	0.46–0.69	0.53–0.94	0.45–0.81	-
Glucagon vs. insulin								
*N* (%, number on total) 38.9 (14/36)		0.0 (0/36)	5.6 (2/36)	13.9 (5/36)	75.0 (27/36)	0.0 (0/36)	47.2 (17/36)	0.0 (0/36)
R^2^ (Mean ± SE) 0.63 ± 0.02		-	-	0.60 ± 0.02	0.80 ± 0.02	-	0.65 ± 0.02	-
R^2^ (Min–Max) 0.47–0.90		-	0.47–0.57	0.50–0.75	0.47–0.96	-	0.50–0.89	-

**Table 5:** Number of fluctuations of glucagon curves in different metabolic tests.

	**Mixed Meal**		**Glucose**		**Protein**		**Fat**	
	**ΔAUC_GLUCA_** **Positive**	**ΔAUC_GLUCA_** **Negative**	**ΔAUC_GLUCA_** **Positive**	**ΔAUC_GLUCA_** **Negative**	**ΔAUC_GLUCA_** **Positive**	**ΔAUC_GLUCA_** **Negative**	**ΔAUC_GLUCA_** **Positive**	**ΔAUC_GLUCA_** **Negative**
Number of fluctuations (%, number on total)								
1	6.3 (2/32)	0.0 (0/4)	0.0 (0/9)	7.4 (2/27)	55.9 (19/34)	0.0 (0/2)	36.7 (4/11)	12.0 (3/25)
2	12.5 (4/32)	25.0 (1/4)	11.1 (1/9)	25.9 (7/27)	2.9 (1/34)	50.0 (1/2)	18.2 (2/11)	24.0 (6/25)
3	31.3 (10/32)	25.0 (1/4)	33.3 (3/9)	48.1 (13/27)	32.4 (11/34)	0.0 (0/2)	18.2 (2/11)	24.0 (6/25)
4	28.1 (9/32)	50.0 (2/4)	33.3 (3/9)	14.8 (4/27)	5.9 (2/34)	50.0 (1/2)	9.1 (1/11)	8.0 (2/25)
5	21.9 (7/32)	0.0 (0/4)	22.2 (2/9)	3.7 (1/27)	0.0 (0/34)	0.0 (0/2)	9.1 (1/11)	20.0 (5/25)

## Data Availability

Data are available upon reasonable request to Prof. Bo Ahrén. They are not publicly available due to data protection concerns related to privacy issues.

## References

[1] Ahrén B. (2015). Glucagon–Early breakthroughs and recent discoveries. Peptides.

[2] Gerich J.E., Schneider V., Dippe S.E., Langlois M., Noacco C., Karam J.H., Forsham P.H. (1974). Characterization of the glucagon response to hypoglycemia in man. J. Clin. Endocrinol. Metab..

[3] Bolli G.B., Dimitriadis G.D., Pehling G.B., Baker B.A., Haymond M.W., Cryer P.E., Gerich J.E. (1984). Abnormal glucose counterregulation after subcutaneous insulin in insulin-dependent diabetes mellitus. N. Engl. J. Med..

[4] Cryer P.E. (1981). Glucose counterregulation in man. Diabetes.

[5] Pipeleers D.G., Schuit F.C., in’t Veld P.A., Maes E., Hooghe-Peters E.L., Van de Winkel M., Gepts W. (1985). Interplay of nutrients and hormones in the regulation of insulin release. Endocrinology.

[6] Sørensen H., Winzell M.S., Brand C.L., Fosgerau K., Gelling R.W., Nishimura E., Ahren B. (2006). Glucagon receptor knockout mice display increased insulin sensitivity and impaired beta-cell function. Diabetes.

[7] Omar B., Sörhede-Winzell M., Ahrén B. (2014). Conditional glucagon receptor overexpression has multi-faceted consequences for beta-cell function. Metabolism.

[8] Gromada J., Chabosseau P., Rutter G.A. (2018). The α-cell in diabetes mellitus. Nat. Rev. Endocrinol..

[9] Guettet C., Rostaqui N., Mathé D., Lecuyer B., Navarro N., Jacotot B. (1991). Effect of chronic glucagon administration on lipoprotein composition in normally fed, fasted and cholesterol-fed rats. Lipids.

[10] Guettet C., Mathé D., Navarro N., Lecuyer B. (1989). Effects of chronic glucagon administration on rat lipoprotein composition. Biochim. Biophys. Acta.

[11] Prip-Buus C., Pegorier J.P., Duee P.H., Kohl C., Girard J. (1990). Evidence that the sensitivity of carnitine palmitoyltransferase I to inhibition by malonyl-CoA is an important site of regulation of hepatic fatty acid oxidation in the fetal and newborn rabbit. Perinatal development and effects of pancreatic hormones in cultured rabbit hepatocytes. Biochem. J..

[12] Briant L., Salehib A., Vergaria E., Zhanga Q., Rorsmana P. (2016). Glucagon secretion from pancreatic a-cells. Upsala J. Med. Sci..

[13] Zhang Q., Ramracheya R., Lahmann C., Tarasov A., Bengtsson M., Braha O., Braun M., Brereton M., Collins S., Galvanovskis J. (2013). Role of KATP channels in glucose-regulated glucagon secretion and impaired counterregulation in type 2 diabetes. Cell. Metab..

[14] Zhang Q., Chibalina M.V., Bengtsson M., Groschner L.N., Ramracheya R., Rorsman N.J., Leiss V., Nassar M.A., Welling A., Gribble F.M. (2014). Na+ current properties in islet α- and β-cells reflect cell-specific Scn3a and Scn9a expression. J. Physiol..

[15] Yu Q., Shuai H., Ahooghalandari P., Gylfe E., Tengholm A. (2019). Glucose controls glucagon secretion by directly modulating cAMP in alpha cells. Diabetologia.

[16] Lund A., Bagger J.I., Christensen M., Knop F.K., Vilsbøll T. (2014). Glucagon and type 2 diabetes: The return of the alpha cell. Curr. Diabetes Rep..

[17] Bonner C., Kerr-Conte J., Gmyr V., Queniat G., Moerman E., Thévenet J., Beaucamps C., Delalleau N., Popescu I., Malaisse W.J. (2015). Inhibition of the glucose transporter SGLT2 with dapagliflozin in pancreatic alpha cells triggers glucagon secretion. Nat. Med..

[18] Ostenson C.G. (1979). Regulation of glucagon release: Effects of insulin on the pancreatic A2-cell of the guinea pig. Diabetologia.

[19] Rorsman P., Berggren P.O., Bokvist K., Ericson H., Möhler H., Ostenson C.G., Smith P.A. (1989). Glucose-inhibition of glucagon secretion involves activation of GABAA-receptor chloride channels. Nature.

[20] Cejvan K., Coy D.H., Efendic S. (2003). Intra-islet somatostatin regulates glucagon release via type 2 somatostatin receptors in rats. Diabetes.

[21] Strowski M.Z., Parmar R.M., Blake A.D., Schaeffer J.M. (2000). Somatostatin inhibits insulin and glucagon secretion via two receptors subtypes: An in vitro study of pancreatic islets from somatostatin receptor 2 knockout mice. Endocrinology.

[22] Vieira E., Salehi A., Gylfe E. (2007). Glucose inhibits glucagon secretion by a direct effect on mouse pancreatic alpha cells. Diabetologia.

[23] Cheng-Xue R., Gómez-Ruiz A., Antoine N., Noël L.A., Chae H.Y., Ravier M.A., Chimienti F., Schuit F.C., Gilon P. (2013). Tolbutamide controls glucagon release from mouse islets differently than glucose: Involvement of K(ATP) channels from both α-cells and δ-cells. Diabetes.

[24] Vergari E., Knudsen J.G., Ramracheya R., Salehi A., Zhang Q., Adam J., Asterholm I.W., Benrick A., Briant L.J.B., Chibalina M.V. (2019). Insulin inhibits glucagon release by SGLT2-induced stimulation of somatostatin secretion. Nat. Commun..

[25] Ahrén B., Taborsky G.J. (1986). The mechanism of vagal nerve stimulation of glucagon and insulin secretion in the dog. Endocrinology.

[26] Ahrén B. (2013). Avoiding hypoglycemia: A key to success for glucose-lowering therapy in type 2 diabetes. Vasc. Health Risk Manag..

[27] Gromada J., Franklin I., Wollheim C.B. (2007). Alpha-cells of the endocrine pancreas: 35 years of research but the enigma remains. Endocr. Rev..

[28] Christensen M., Vedtofte L., Holst J.J., Vilsbøll T., Knop F.K. (2011). Glucose-dependent insulinotropic polypeptide: A bifunctional glucose-dependent regulator of glucagon and insulin secretion in humans. Diabetes.

[29] Ohlsson L., Alsalim W., Carr R.D., Tura A., Pacini G., Mari A., Ahrén B. (2013). Glucose-lowering effect of the DPP-4 inhibitor sitagliptin after glucose and non-glucose macronutrient ingestion in non-diabetic subjects. Diabetes Obes. Metab..

[30] Alsalim W., Tura A., Pacini G., Omar B., Bizzotto R., Mari A., Ahrén B. (2016). Mixed meal ingestion diminishes glucose excursion in comparison with glucose ingestion via several adaptive mechanisms in people with and without type 2 diabetes. Diabetes Obes. Metab..

[31] Mari A., Tura A., Gastaldelli A., Ferrannini E. (2002). Assessing insulin secretion by modeling in multiple-meal tests: Role of potentiation. Diabetes.

[32] Tura A., Muscelli E., Gastaldelli A., Ferrannini E., Mari A. (2014). Altered pattern of the incretin effect as assessed by modelling in individuals with glucose tolerance ranging from normal to diabetic. Diabetologia.

[33] Gastaldelli A., Abdul Ghani M., DeFronzo R.A. (2021). Adaptation of Insulin Clearance to Metabolic Demand Is a Key Determinant of Glucose Tolerance. Diabetes.

[34] Matsuda M., De Fronzo R.A. (1999). Insulin sensitivity indices obtained from oral glucose tolerance testing: Comparison with the euglycemic insulin clamp. Diabetes Care.

[35] de Toro-Martín J., Arsenault B.J., Després J.P., Vohl M.C. (2017). Precision Nutrition: A Review of Personalized Nutritional Approaches for the Prevention and Management of Metabolic Syndrome. Nutrients.

[36] Chung W.K., Erion K., Florez J.C., Hattersley A.T., Hivert M.F., Lee C.G., McCarthy M.I., Nolan J.J., Norris J.M., Pearson E.R. (2020). Precision Medicine in Diabetes: A Consensus Report from the American Diabetes Association (ADA) and the European Association for the Study of Diabetes (EASD). Diabetes Care.

[37] Yabe D., Kuroe A., Watanabe K., Iwasaki M., Hamasaki A., Hamamoto Y., Harada N., Yamane S., Lee S., Murotani K. (2015). Early phase glucagon and insulin secretory abnormalities, but not incretin secretion, are similarly responsible for hyperglycemia after ingestion of nutrients. J. Diabetes Its Complicat..

[38] Bergman M., Abdul-Ghani M., DeFronzo R.A., Manco M., Sesti G., Fiorentino T.V., Ceriello A., Rhee M., Phillips L.S., Chung S. (2020). Review of methods for detecting glycemic disorders. Diabetes Res. Clin. Pract..

[39] Tura A., Morbiducci U., Sbrignadello S., Winhofer Y., Pacini G., Kautzky-Willer A. (2011). Shape of glucose, insulin, C-peptide curves during a 3-h oral glucose tolerance test: Any relationship with the degree of glucose tolerance?. Am. J. Physiol. Regul. Integr. Comp. Physiol..

[40] Morettini M., Burattini L., Göbl C., Pacini G., Ahrén B., Tura A. (2021). Mathematical Model of Glucagon Kinetics for the Assessment of Insulin-Mediated Glucagon Inhibition During an Oral Glucose Tolerance Test. Front. Endocrinol..

